# Development and psychometric properties of a scale for measuring internal participation from a patient and health care professional perspective

**DOI:** 10.1186/1472-6963-13-374

**Published:** 2013-10-01

**Authors:** Mirjam Körner, Markus A Wirtz

**Affiliations:** 1 Medical Psychology and Medical Sociology, Medical Faculty, University of Freiburg, Hebelstr. 29, 79104 Freiburg, Germany; 2 Department of Research Methods, Institute of Psychology, University of Education Freiburg, Kunzenweg 21, 79117 Freiburg KG IV 204B Germany

**Keywords:** Internal participation, Interprofessional collaboration, Team, Psychometrics, Scale development, Healthcare, Germany

## Abstract

**Background:**

Effective patient-centred health care requires internal participation, which is defined as interprofessional patient-centred teamwork. Many scales are designed for measuring teamwork from the perspective of one type of health care professional (e.g. physician or nurse), rather than for the use for all health care professionals as well as patients. Hence, this paper’s purpose is to develop a scale for measuring internal participation from all relevant perspectives and to check its psychometric properties.

**Methods:**

In a multicentre cross-sectional study, a 6-item Internal Participation Scale (IPS) was developed and administered to 661 health care professionals (staff) and 1419 patients in 15 rehabilitation clinics to test item characteristics, acceptance, reliability (internal consistency) and construct validity. Additionally, we performed an exploratory factor analysis (EFA) to determine the factorial structure and explained variance. Confirmatory factor analysis (CFA) was used to verify the theoretically assumed one-dimensional factorial structure.

**Results:**

A total of 275 health care professionals and 662 patients participated, and the complete data sets of 272 staff members and 536 patients were included in the final analysis. The discrimination index was above .4 for all items in both samples. Internal consistency was very good, with Cronbach’s alpha equalling .87 for the staff and .88 for the patient sample. EFA supported a one-dimensional structure of the instrument (explained variance: 61.1% (staff) and 62.3% (patients)). CFA verified the factorial structure, with the factor loadings exceeding .4 for five of six items in both samples. Global goodness-of-fit indices indicated a good model fit, with a Tucker-Lewis index (TLI) of .974 (staff) and .976 (patients) and a comparative fit index (CFI) of .988 (staff) and .989 (patients). The root mean square error of approximation (RMSEA) amounted to .068 for the patient sample and .069 for the staff sample. There is evidence of construct validity for both populations.

**Conclusions:**

The analysis of the scale’s psychometric properties resulted in good values. The scale is a promising instrument to assess internal participation from the perspective of both patients and staff. Further research should investigate the scale’s psychometric properties in other interprofessional health care settings to examine its generalizability as well as its sensitivity to change.

## Background

**Figure 1:**
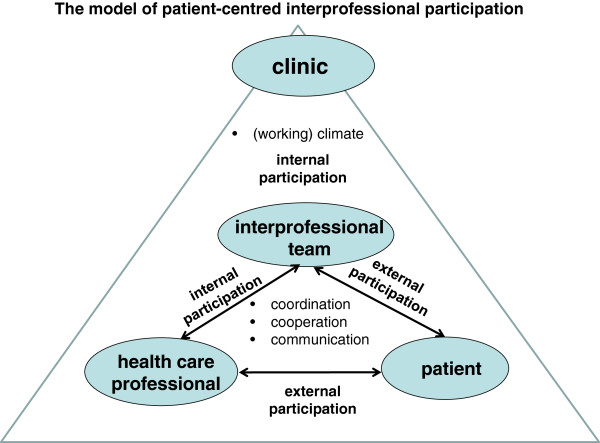
The model of patient-centred interprofessional participation.

Patient-centredness is one of the most essential quality and outcome criteria in health care. The concepts about the dimensions of patient-centredness varied widely [1-7]. Some focus exclusively on the patient and physician and their interaction [5-7], while broader concepts include structural and organizational aspects such as access to care, coordination and continuity, information technology, interprofessional teamwork, etc. [2,4,8,9]. Based on the model of integrated patient-centredness [8,9], the core dimension of patient-centredness is participation – patient participation in the encounter between patient and health care professional (external participation) as well as participation within the interprofessional team of health care professionals (internal participation) [8,9]. Both participation forms are described through four Cs – communication, cooperation, coordination, and (working) climate (see Figure 1).

Internal participation is a key factor to increase the effectiveness of health care services [10]; it can help to enhance patient-centredness, patient safety and successful treatment [11-17] and is associated with improved patient satisfaction [17-19] and employee satisfaction [11] as well as cost savings [11,20].

The term *internal participation* is used to describe teamwork, e.g. goal setting, negotiation of roles, leadership, and shared decision-making in health care settings [21]. The aim of internal participation is to create a partnership between health care professionals “in a participatory, collaborative and coordinated approach to shared decision making around health and social issues” of the patients. [11], p. 11]. Hence, *internal participation* is defined as teamwork between two or more health care professionals from different disciplines to provide comprehensive services to patients, or in other words interprofessional patient-centred teamwork [9,22-25].

Several instruments are available to assess teamwork in health care settings [20,26]. However, none of them have been specifically developed for interprofessional patient-centred teamwork, and none include the patients’ perspective. For example, the current review by Valentine et al. [20] identified and described 39 surveys designed to measure teamwork, whereof only ten met all criteria of psychometric validity. Among these ten, none measured the dimensions of internal participation with a short scale from the perspectives of health care professionals and patients in health care settings such as rehabilitation clinics.

Medical rehabilitation in Germany mostly takes place in an inpatient setting, where multiple health care professionals (physician, psychologist, occupational therapist, physical therapist, nurse, social worker, etc.) work together in a team to deliver comprehensive patient care. Measuring the patient’s opinion of internal participation is important based on the idea that the patient is an equal partner in treatment and should be involved as a partner in the treatment communication and coordination process [27]. This is substantiated by the fact that in some fields of medicine (e.g. psychosomatics, oncology, and addiction), patients and their families are even involved as members of the treatment team. In addition, the fact that the evaluation by different health care professionals as well as internal evaluation (staff) and external evaluation (patients) often differ significantly underpins the relevance of assessing multiple perspectives. Different perspectives may complement each other to reveal a valid impression of the general situation or document unique characteristics of stakeholder perspectives, respectively. The main benefit is that the stakeholder perspectives could be used as basic information for intervention programmes, such as team development or interprofessional training to enhance patient-orientation.

Since the daily practice in health care organizations is very hectic and internal participation is only one part of measuring patient-centredness, the instrument should be extremely brief, a short scale with five to ten items.

Based on these assumptions, the aim of the study was to develop and psychometrically test a brief instrument (short scale) for measuring internal participation in interprofessional health care settings from a patient and staff perspective.

## Methods

### Study design and population

This study was part of the project “Development and evaluation of a shared-decision-making training program in medical rehabilitation”, which is a multi-centre cluster-randomised controlled study. For the purpose of this study, we used the patient and staff questionnaires of the first data collection period (cross-sectional data).

Twenty-two inpatient medical rehabilitation clinics in southwest Germany originally expressed interest in the study; of these, fifteen took part in both surveys. Each clinic determined a contact person responsible for the study process: all surveys were then sent to this contact person (mostly senior physician or psychologist), who distributed them to the patients at the end of their stay in the rehabilitation clinic and to health care professionals in the treatment team. We only had one contact to patients and sent out one reminder to health care professionals working at the clinics two weeks after the deadline for returning questionnaires.

Inclusion criteria for patients were: chronic disease (somatic or psychosomatic), treated at one of the rehabilitation clinics, age of 18 years or older, sufficient German language abilities, no cognitive impairments and written informed consent. Inclusion criteria for the staff were: health care professional (e.g. physician, nurse, physical therapist, sports teacher, masseur, occupational therapist, psychologist or other psychosocial therapist, dietician or social worker), involvement in a treatment team in the inpatient rehabilitation clinics and direct participation in patient treatment.

The study was approved by the Ethics Committee of the University of Freiburg without any ethical concerns.

### Instruments

#### Internal participation scale (IPS)

The Internal Participation Scale (IPS) was developed based on theory. The items were selected based on the key dimensions of participation (communication, coordination, cooperation, and climate) in the model of patient-centred interprofessional participation, which is the main part of the model of integrated-patient-centredness [8,9]. The literature on teamwork assessment underpins the importance of these dimensions [20,26]. In the review by Valentine et al. [20], the core dimensions of existing English-language teamwork assessments for health care are communication, coordination, and respect. Therefore we added “respect” as a further dimension of internal participation to the scale with one item. In addition, the development process took into account the two fundamental elements of team functioning in team models (e.g. the model of “team reflexivity”, the Kassel team pyramid) and teamwork questionnaires (e.g. Team Reflexivity Questionnaire, Questionnaire on Teamwork) – task-specific and social elements [28,29]. Both dimensions proved to be important for high team functioning and team development. Since common tasks can only be managed through effective and efficient patient-centred communication, cooperation and coordination among the different health care professionals, we first developed items to measure these task-oriented aspects of internal participation (items 2–5). To assess the entire spectrum of internal participation two items measuring social-oriented aspects - respect (item 6) and working climate (item 1) – have been newly developed to complete the scale. The final items and their short labels are listed in Table 1.

**Table 1:** Items of the IPS (staff and patient version)

**Topics (short labels)**		**Items**
**Climate**	[1]	Overall there is a friendly climate in the clinic.
**Cooperation**	[2]	The health care professionals work hand-in-hand
**Agreements**	[3]	Agreements made amongst health care professionals are well coordinated.
**Coordination**	[4]	The different types of treatment are well coordinated.
**Communication**	[5]	Communication in the team is efficient.
**Respect**	[6]	The health care professionals respect each other.

The IPS was designed for use in patient and in staff surveys. Therefore, its language should be appropriate for both groups. The selection and wording of the items of the IPS were the result of a discussion and consensus process in which three health care research experts (university-based staff of the project) engaged, with feedback from two rehabilitation experts (one quality manager and one senior physician) and three persons with chronic diseases (different ages, genders, and education) with experience as rehabilitation patients.

The items of the scale were rated on a four-point Likert scale, ranging from 1 (does not apply at all), to 2 (does not generally apply), 3 (generally applies) and 4 (fully applies), with the further possibility to tick a box “I can’t judge this”. The scale value was calculated as the mean of the six items and transformed to a scale from 0 (minimal participation) to 100 (maximal participation). When calculating the total score (team score), one missing item was accepted. If more than one item was missing, no total score was calculated.

The items of the IPS were translated from German^a^ into English by a bilingual professional translator and then translated back by another bilingual professional translator. Then the original German version was compared with the back-translated version. After discussion of the differences in the items, the final English version was generated. The result was again provided to a bilingual speaker for translation into German to confirm the accuracy of the translation process.

#### Instruments for testing validity

For testing discriminant validity of the IPS, we used the construct “external participation” measured by the *9-item Shared Decision Making Questionnaire* (*SDM-Q-9)*[30] in the patient survey and the *Shared Decision Making Questionnaire* – *physician version (SDM-Q-Doc)*[31] in the staff survey. They are both standardised brief instruments (9 items) for assessing shared decision-making (external participation) in clinical encounters. Both instruments were originally developed for physicians and were adapted to all health care professionals in our study. Item and scale characteristics were tested in the study and were comparable with the original version. Furthermore, “health status” (*IRES-24)*[32] was used for determining discriminant validity for patient data.

For defining convergent validity, the standardised *Questionnaire on staff satisfaction in medical rehabilitation* was used [33,34]. It consists of three scales: *workplace atmosphere/climate* (7 items), *leadership* (14 items) as well as *organization and communication* (10 items). All three scales show good to acceptable reliability [33].

To test convergent validity for the patient population, the established and validated *Questionnaire on Patient Satisfaction*[35,36] was applied. It consists of eight items and is based on the American “Client Satisfaction Questionnaire CSQ-8” [37].

### Statistical analysis

Before analysis, quality was tested by verification of random samples, the items were checked for plausibility, and missing data analysis was performed. If there was more than one missing item in the IPS or more than 30% of items missing in one whole data set of one patient or health care professional, the data set was excluded; otherwise, data was imputed by means of the expectation maximisation algorithm, which is one of the recommended methods to avoid bias even in cases of data missing at random (MAR) [38].

Descriptive statistics were calculated using SPSS version 20.0, and structural equation modelling (SEM) was carried out using the AMOS software version 20.0 (maximum likelihood method). Acceptance (completion rate of the items in per cent), discrimination (corrected item-total correlation), and difficulty (mean) were used for all items in both samples to describe item characteristics. The reliability of the IPS was measured by calculating internal consistency (Cronbach’s alpha), which is a commonly used statistical parameter. For newly developed scales or surveys, a minimum of .70 Cronbach’s alpha is generally considered acceptable. A good value of Cronbach’s alpha is between .70 and .90. [39-42]. Additionally, the item characteristics were calculated for different subgroups based on organizational (indication field) and demographic characteristics (gender, education, profession, and age) to identify potential differential item functioning.

After conducting an exploratory factor analysis (EFA) to determine the factorial structure and the explained variance of the construct *internal participation*, confirmatory factor analysis (CFA, structural equation modelling, SEM with AMOS 20.0) was used to verify the theoretically assumed one-dimensional structure. Several global fit measures have been calculated to determine whether the empirical associations are in accordance with the proposed one-dimensional model assumption, normed chi-squared (*χ*^2^/df), comparative fit index (CFI), Tucker-Lewis index (TLI), and Root Mean Square Error of Approximation (RMSEA) were used as Goodness-of-fit indicators. The *χ*^2^-test is the strictest form of model testing [40] as it tests the equality of information assumed by the model and measured in the empirical covariance matrix. For the normed chi-squared value, a cut off value of ≤ 2.5 is recommended [43]. However, both measures depend critically on sample size, and may lead to inappropriate high power especially in larger samples (N > 300) [43]. Therefore measures of the approximate model fit have been developed. The Root Mean Square Error of Approximation (RMSEA) is the proportion of variance-covariance information not correctly predicted by the model, with values of ≤ .08 indicating an acceptable fit and values ≤ .05 a good fit [44]. In addition the Tucker-Lewis Index (TLI) and the Comparative Fit Index (CFI) were calculated. For these measures, values ≥ .90 are suggested for an acceptable model fit and ≥ .95 for a good model fit [44,45]. Indicators of local fit were also applied. The proportion of variance of the indicator reliability (IR) explained by the construct should amount to > .40, and the average proportion of variance (AVE) measured by the construct should be > .50 [40]. As criterion for factor reliability (FR), values > .60 are accepted as satisfactory [46].

Besides these analyses, we examined construct validity of the IPS. The association with similar (convergent validity) and different scales (discriminant validity) was tested using bivariate analysis (Pearson’s correlation coefficient). For discriminant validity, a weak association (r ≤ .4) is expected between the constructs internal participation (IPS) and external participation (SDM-Q-9 [30] or SDM-Q-Doc [31]) and health status (IRES-24 [32])), and for convergent validity, a high association (r ≥ .6) is expected between the constructs internal participation (IPS) and satisfaction (staff: Questionnaire on staff satisfaction in medical rehabilitation [33]; patients: Questionnaire on patient satisfaction [35,36]).

## Results

### Sample characteristics of patients

A total of N = 1419 questionnaires were sent out, with N = 662 filled out and returned (response rate = 46.6%). After missing data analysis, a total of 536 complete data sets were included in the current analysis. Table 2 provides a description of the patient sample and shows that more men than women completed the questionnaire. The average age of the patient sample was 52.7 years. The majority of respondents was German and had a low educational level. Almost half of the patients were employed, and more than half were married. Nearly two thirds had undergone treatment in somatic rehabilitation clinics and slightly more than one third in psychosomatic rehabilitation clinics. Of the psychosomatic patients, 25% were in rehabilitation because of addiction. Slightly more than one fourth had suffered from their illness for more than ten years (see Table 2).

**Table 2:** Description of patient sample (n = 536)

	***Frequency***	***Per cent***
**Gender**		
Male	334	62.3
Female	198	36.9
Missing	4	.7
**Age**		
Mean 52.7 (SD = 13.7, range: 18–90)		
**Nationality**		
German	499	93.1
Other Nationalities	32	6.0
Missing	5	.9
**Education**		
Low	233	43.5
Medium	177	33.0
High	106	19.8
Other	12	2.2
Missing	8	1.5
**Occupation**		
Employed	256	47.8
Retired	132	24.6
Homemaker	24	4.5
Unemployed	92	17.2
Other	24	4.5
Missing	8	1.5
**Family status**		
Never married	112	20.9
Married	279	52.1
Divorced	102	19.0
Widowed	37	6.9
Missing	6	1.1
**Indication fields**		
Psychosomatic (mental health problems)	182	34.0
Somatic	346	64.6
- Orthopedics	127	23.7
- Oncology	70	13.1
- Neurology	48	9.0
- Cardiology	20	3.7
- Other somatic diseases	81	15.1
Missing	8	1.4
**Duration of illness**		
Less than six months	87	16.2
More than six to 12 months	85	15.9
More than one year to two years	62	11.6
More than two to five years	80	14.9
More than five to ten years	76	14.2
More than ten years	140	26.1
Missing	6	1.1

Comparing gender and age of the patient sample with the statistics of the German statutory pension insurance scheme [47] revealed significant differences. When compared to all rehabilitation patients in Germany, the proportion of men in the sample is higher (by approximately 10%). Furthermore, the average age of study patients is three years higher than that of all patients in Germany whose rehabilitation is paid by the German statutory pension insurance scheme [47].

### Sample characteristics of staff

Of 661 questionnaires sent out to staff of the health care teams in the medical rehabilitation clinics, 275 were returned (rate of return: 41.6%). Three questionnaires were excluded because too much information was missing (more than 30% of the total questionnaire), resulting in a total of 272 staff surveys that could be analysed. Table 3 displays the sample characteristics of the staff survey. In contrast to the patient survey, more females (60.3%) than males (34.6%) participated here. Most of the health care professionals were in the age groups ’36 to 45’ (30.1%) and ’46 to 55’ (32.4%), worked full time, and had worked more than five years in the clinic.

**Table 3:** Description of staff sample (n = 272)

	***Frequency***	***Per cent***
**Gender**		
Male	94	34.6
Female	164	60.3
Missing	14	5.1
**Age Groups**		
17-25	12	4.4
26-35	40	14.7
36-45	82	30.1
46-55	88	32.4
56-65	38	14.0
Missing	12	4.4
**Professionals**		
Physicians	49	18.0
Nursing staff	48	17.6
Psychosocial therapists	67	24.6
Physical therapists	50	18.4
Others	37	13.6
More than one professional group	12	4.4
Missing	9	3.3
**Job tenure**		
More than one year, but less than three years	37	13.6
Three to five years	26	9.6
More than five years	190	69.9
Less than one year	13	4.8
Missing	6	2.2
**Employment**		
Full-time	174	64.0
Part-time (more than 70% but less than 100%)	41	18.0
Part-time (more than 30% but less than 70%)	35	15.1
Missing	14	2.9

#### Item characteristics and reliability of IPS

Table 4 shows the completion rates of IPS as an indicator of acceptance, item difficulty, and item discriminations. The patient completion rate ranged between 85.1% and 99.3%, with the staff rate slightly higher (93.6% to 99.6%). The mean of the items (difficulty) ranged from 3.36 to 3.63 for patients and 2.84 to 3.22 for the staff on a scale from 1 (does not apply at all) to 4 (fully applies). Corrected item-total correlation (discrimination) was above .4 for all items in both samples, while internal consistency (Cronbach’s alpha) was .878 for patients and .871 for staff. In the exploratory factor analysis, all six items loaded on one factor (explained variance: 61.1% staff, 62.3% patient).

**Table 4:** Item characteristics of the IPS in both versions

**Item**		**Acceptance (completion rates in per cent)**		**Difficulty (mean; range 1–4)**		**Discrimination (corrected item-total correlation)**	
		**Patient**	**Staff**	**Patient**	**Staff**	**Patient**	**Staff**
1	climate	99.3	99.3	3.63	3.22	.622	.759
2	cooperation	94.4	96.7	3.48	2.93	.794	.788
3	agreements	89.9	94.9	3.45	2.91	.759	.773
4	coordination	97.2	93.6	3.36	2.84	.715	.653
5	communication	85.1	99.6	3.47	3.13	.816	.794
6	respect	87.7	99.6	3.58	2.89	.705	.788

Inter-item correlations for the patient survey ranged from .377 to .733 and for the staff survey from .349 to .686 (see Table 5).

**Table 5:** Inter-item correlations (patient/staff survey)

	***Item 1: *climate**	***Item 2: *cooperation**	***Item 3: *agreements**	***Item 4: *coordination**	***Item 5: *communi-cation**	***Item 6: *respect**
*Item 1:* climate	1	.533	.413	.380	.463	.377
*Item 2:* cooperation	***.606***	1	.733	.524	.637	.489
*Item 3:* agreements	***.508***	***.679***	1	.523	.639	.489
*Item 4:* coordination	***.349***	***.438***	***.456***	1	.568	.488
*Item 5:* communi-cation	***.580***	***.511***	***.521***	***.400***	1	.638
*Item 6:* respect	***.534***	***.518***	***.544***	***.434***	***.686***	1

NOTE:

Staff survey results are printed bold and Italics in the lower off-diagonal cells.

All correlations are significant at p ≤ 0.001.

#### Characteristics of the IPS for subgroups

Table 6 summarises the item characteristics of the IPS for subgroups in both populations, patient and staff. The patient analyses show a discrimination coefficient > .4 for all subgroups. Most of the discrimination coefficients in the staff subgroups are also above .4, with only two below: for item 5 (communication item) in both cases. The difficulty of the items ranges from 2.56 to 3.98, and internal consistency measured by Cronbach’s alpha is higher than .8, except in psychosocial therapists. For the physicians and age group 56 years and older, it is above .9.

**Table 6:** Item characteristics of the IPS in subgroups of patients and staff

**Subgroup**	**N**	**Discrimination (corrected item-total correlation) range**	**Difficulty (mean; range 1–4)**	**Internal consistency (Cronbach’s alpha)**
**Patients**				
**Indication group**				
- Somatic	345	.556-.770	3.43-3.72	.878
- Psychosomatic	191	.473-.763	3.21-3.47	.853
**Gender**				
- Male	334	.549-.785	3.40-3.58	.875
- Female	198	.537-.787	3.40-3.71	.882
**Education**				
- Low	233	.536-.799	3.51-3.67	.864
- Medium	177	.556-.802	3.32-3.60	.898
- High	106	.558-.721	3.29-3.58	.846
**Age groups**				
- 18-40	97	.414-.757	3.31-3.51	.855
- 41-60	276	.540-.772	3.35-3.59	.865
- >60	151	.515-.775	3.67-3.83	.867
**Staff**				
**Indication group**				
- Somatic	198	.506-.736	2.92-3.31	.872
- Psychosomatic	52	.510-.713	2.56-2.96	.834
**Gender**				
- Male	94	.644-.791	2.82-3.12	.898
- Female	164	.670-.741	2.83-3.27	.856
**Professionals**				
- Physicians	49	.741-.781	3.00-3.47	.916
- Nursing staff	48	.294-.758	2.63-3.08	.871
- Psychosocial therapists	67	.356-.567	2.73-3.98	.746
- Physical therapists	50	.444-.816	2.88-3.41	.867
- Other professionals	37	.507-.715	2.74-3.32	.824
**Age groups**				
- 17 – 35	52	.480-.732	2.76-3.46	.806
- 36 - 55	170	.504-.775	2.84-3.15	.883
- 56 and older	38	.675-.868	2.88-3.24	.906

#### Model fit

**Figure 2:**
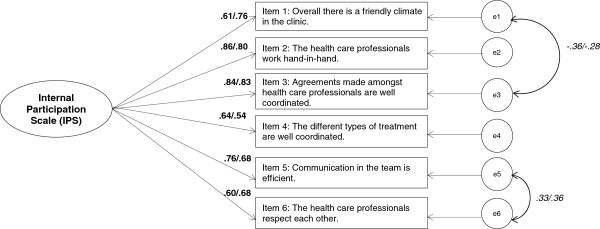
**The confirmatory factor model of the Internal Participation Scale (IPS).** Bold printed figures are the factor loadings, first figure = value of patient version; second figure (behind the slash) = value of staff version. The figures in Italics are the error correlation: first figure = value of patient version; second figure (behind the slash) = value of staff version.

The model fit indices in Table 7 indicate that in part, the data insufficiently fit the original model (see especially RMSEA, row original model). Local dependencies between items 1 (climate) and 3 (agreements) and between items 5 (communication) and 6 (respect) were the sources of this problem. Considering these two local dependencies (error correlations, see Figure 2), the thresholds for an acceptable to good model fit was reached. Global goodness-of-fit indices of the modified model showed a good model fit with a normed *χ*^2^ of 2.241 for the staff, but not for the patient sample (normed *χ*^2^ = 3.526). Excellent model fit is reached with a TLI of .974 for staff and .976 for patients and a CFI of .988 and .989, respectively (see Table 7). RMSEA is .069 for the patient survey and .068 for the staff survey.

**Table 7:** Global fit indices for all estimated models

		***X***^**2**^	**df**	**p**	***X***^**2**^**/df**	**TLI**	**CFI**	**RMSEA []**
Original Model	Health care professionals (staff)	58.597	9	<.001	6.511	.885	.931	[.109**, .143**, .178]
	Patients	77.921	9	<.001	8.658	.928	.957	[.096**, .120**, .145]
Modified Model	Health care professionals (staff)	15.684	7	.028	2.241	.974	.988	[.021, **.068**, .113]
	Patients	24.685	7	<.001	3.526	.976	.989	[.041, **.069**, .099]

Note: bold = sample estimate.

Despite these local dependencies, the factor loadings of the items on the IPS were high (see Figure 2: final confirmatory factor model). In total, factor loadings exceed .4 for five of six items in both populations. Item 4 (coordination) (.29) in the staff sample and item 1 (climate) (.37) in the patient sample had somewhat lower loadings (see Table 8). The average variance explained is .54 for both samples, and factor reliability is also good for both (.87).

**Table 8:** Measures of local fit for the modified CFA model

**Item**		**Indicator reliability (IR)**		**Critical ratio (CR)**		**Factor reliability (FR)**		**Average variance extracted (AVE)**	
		**Patients**	**Staff**	**Patients**	**Staff**	**Patients**	**Staff**	**Patients**	**Staff**
1	climate	.37	.58	1^1)^	1^1)^	.87	.87	.54	.54
2	cooperation	.74	.64	1.679***	.957***				
3	agreements	.72	.69	1.643***	1.066***				
4	coordination	.42	.29	1.361***	.608***				
5	communication	.63	.47	1.500***	.855***				
6	respect	.40	.46	1.071***	.989***				

Note: ***p < 0 .001, ** p < .01; ^1)^ Fixed to 1 to ensure identifiability.

#### Construct validity of IPS

There was evidence of validity since the scales correlated highly with the related but independent indicators for teamwork. The IPS correlated significantly with organization and communication with a correlation coefficient of .578, with workplace atmosphere/climate to .748, and leadership appraisal to .551 in the staff survey. There was also a significant high correlation (.593) for IPS with patient satisfaction in the patient survey. In contrast, correlation with the non-related individual items was low. Internal participation showed significant low correlation with external participation in both surveys (staff: .249, patients: .262), and there was no association (r = .039) with health status in the patient survey (see Table 9).

**Table 9:** Convergent and discriminant validity of IPS (bivariate correlation coefficients)

		**Correlation with IPS**
	***Staff questionnaire***	
Convergent validity	Organization and communication ^1)^	.578**
	Workplace Atmosphere/Climate ^1)^	.748**
	Leadership appraisal ^1)^	.551**
Discriminant validity	External participation (SDM-Q-doc) (staff questionnaire)	.249**
	***Patient survey***	
Convergent validity	Patient satisfaction ^2)^	.593**
Discriminant validity	External participation (SDM-Q-9)	.262**
	Health status (IRES-24)	.039

NOTE: ** Correlations are significant at p ≤ 0.001.

^1)^Questionnaire on staff satisfaction in medical rehabilitation.

^2)^Questionnaire on patient satisfaction (based on the Client Satisfaction Questionnaire).

## Discussion

Within the context of this study, we developed a short scale for assessing internal participation as seen from the patients’ and staff members’ perspective. The study tested the psychometric properties of the self-compiled IPS and analysed the theoretically assumed one-dimensional structure using a confirmatory factor analysis (CFA). The psychometric properties and reliability are good, and construct validity is evident. The data showed acceptable to good model fit for the modified model, which includes the local dependencies between items 1 (climate) and 3 (agreements), and items 5 (communication) and 6 (respect). However, the correlations between these two pairs of items are low in comparison to the factor loadings. These as well as very good indicator reliability (IR) confirm the fit of the items to the construct *internal participation.*

The IPS can be recommended for measuring internal participation in an interprofessional health care setting from both the patient and staff point of view. It is a very clear, simple, and effective instrument, and it includes the three most important aspects of teamwork measurement according to Valentine et al. [20], which are the core competencies of interprofessional collaboration [10,48].

Its suitability for measuring patient and staff perception of internal participation in a health care setting is an innovative feature in comparison to other scales [20,26]. To the best of our knowledge, there is no existing scale that measures internal participation from the perspectives of all health care professionals and patients. Including both perspectives can reveal different perceptions. The scale can therefore be applied to identify sub-cultures (e.g., among professional groups) [49-51]. For example, physicians evaluated internal participation more positively than the other health care professionals [50]. From previous studies on teamwork in rehabilitation, we know that these other health care professionals are often not equal partners in the team. Traditionally, there is a strict hierarchical structure in rehabilitation clinics with the physicians being the leaders. In addition, teams often have more of a multidisciplinary team approach (hierarchical and discipline-oriented organization, one-way and mostly bilateral communication, authoritarian leadership, autonomous decision-making) than an interdisciplinary team approach (patient-centred organization, participative leadership, mutual and multilateral communication, and shared decision-making in the team), despite the fact that the latter is more effective [52,53]. Team interventions could be recommended if there is a high variance between the evaluation results of the different health care professionals in the team.

Due to the economics and simplicity of the short scale, the IPS only measures the most relevant aspects of participation (communication, coordination, cooperation, respect and climate) [20]. The instrument is theory-based [8,9] and offers very good reliability and validity. The clarity of the construct and the quality of the short scale are scientifically proven.

### Limitations

Some limitations must be considered. Representativeness is limited by the absence of data on non-responders and of general data on the patients of the study clinics; therefore we conducted a comparison with the data of the German statutory pension insurance scheme, which revealed differences concerning average age and gender. Generalizability may be limited by a possible self-selection bias due to the voluntary participation of the clinics, patients and staff. It can be assumed that we were only able to reach motivated clinics, staff and patients. Further selection effects are possible with relation to patients since these were recruited within the rehabilitation clinics.

The staff in some clinics had serious doubts regarding the anonymity of the survey. This could be one explanation for the low but acceptable response rate.

As a further limitation of the study, the questionnaire was not pre-tested. Hence, we cannot make any statements about the quality of the wording of the items, e.g. their readability, comprehensibility or interpretation by patients and staff members. Yet, the survey was conducted without problems, and the participants of the study had no difficulties completing the questionnaire.

The lower acceptance rate especially for the items agreements (item 3), respect (item 6), and communication (item 5) in the patient version (these items were answered more often than others with “I can’t judge this”) indicate that evaluating the internal participation aspects is more difficult for patients than for the health care professionals. This shows a lack of involvement in the team for these patients [27].

Our findings concerning validity are limited as a result of using only one method (one questionnaire) to assess all constructs.

Concerning the fit indices, the normed chi-squared value exceeds the critical value (*χ*^2^/df > 2.5). All other values indicate good to excellent model fit. Because the normed chi-squared depends critically on sample size, it is recommended to use predominantly TLI, CFI, and RMSEA for the interpretation of model fit if the sample size is higher than 300 [43]. Therefore, this variance is negligible, and the model fits well.

It should also be mentioned that the measurement characteristics are not spontaneously transferable across languages. The results are specific to the German versions of the instrument, making further evaluation of the psychometric properties of the English version essential. The psychometric properties should also be tested in other settings, for example at university hospitals. Further evaluation should also be focused on generalizability of the IPS, underpinning the need for the scale to be applied in other health care settings, especially in acute care hospitals. Furthermore, the IPS should also be tested to determine if the instrument can assess pre-post changes (sensitivity to change).

## Conclusions

The Internal Participation Scale is the first short scale which allows measuring patient-centred interprofessional teamwork from the perspectives of all health care professionals as well as patients. It is brief and suitable for use in interprofessional health care settings. The item and scale characteristics are good to excellent.

## Endnotes

^a^ The German version of the Internal Participation Scale can be requested by the first author of this article.

## Abbreviations

(AEV): Average variance extracted; (CFA): Confirmatory factor analysis; (CFI): Comparative fit index; (CR): Critical ratio; (df): Degree of freedom; (FR): Factor reliability; (IR): Indicator reliability; (IRES-24): Indicators of rehabilitation status questionnaire; (IPS): Internal participation scale; (MAR): Missing at random; (RMSEA): Root mean square error of approximation; (SEM): Structural equation modeling; (SDM): Shared decision-making; (SDM-Q9): 9-item shared decision making questionnaire; (SDM-Q-9-doc): Shared decision making questionnaire - physician version; (TLI): Tucker-Lewis index; (X2): Chi-squared value, (*X*^2^/df), normed Chi-squared value.

## Competing interests

The authors declare that they have no competing interests.

## Authors’ contributions

MK – Conception and design of the study, data analysis, interpretation of the data, drafting and revising the manuscript, final approval. MW – data analysis, interpretation of the data, revising the manuscript, final approval. Both authors read and approved the final manuscript.

## Pre-publication history

The pre-publication history for this paper can be accessed here:

http://www.biomedcentral.com/1472-6963/13/374/prepub
